# Antagonistic Parent-Offspring Co-Adaptation

**DOI:** 10.1371/journal.pone.0008606

**Published:** 2010-01-06

**Authors:** Mathias Kölliker, Benjamin J. Ridenhour, Sabrina Gaba

**Affiliations:** 1 Zoological Institute, University of Basel, Basel, Switzerland; 2 Department of Biology, University of Idaho, Moscow, Idaho, United States of America; 3 INRA, UMR1210 Biologie et Gestion des Adventices, Dijon, France; University of Utah, United States of America

## Abstract

**Background:**

In species across taxa, offspring have means to influence parental investment (PI). PI thus evolves as an interacting phenotype and indirect genetic effects may strongly affect the co-evolutionary dynamics of offspring and parental behaviors. Evolutionary theory focused on explaining how exaggerated offspring solicitation can be understood as resolution of parent-offspring conflict, but the evolutionary origin and diversification of different forms of family interactions remains unclear.

**Methodology/Principal Findings:**

In contrast to previous theory that largely uses a static approach to predict how “offspring individuals” and “parental individuals” should interact given conflict over PI, we present a dynamic theoretical framework of antagonistic selection on the PI individuals *obtain/take* as offspring and the PI they *provide* as parents to maximize individual lifetime reproductive success; we analyze a deterministic and a stochastic version of this dynamic framework. We show that a zone for equivalent co-adaptation outcomes exists in which stable levels of PI can evolve and be maintained despite fast strategy transitions and ongoing co-evolutionary dynamics. Under antagonistic co-adaptation, cost-free solicitation can evolve as an adaptation to emerging preferences in parents.

**Conclusions/Significance:**

We show that antagonistic selection across the offspring and parental life-stage of individuals favors co-adapted offspring and parental behavior within a zone of equivalent outcomes. This antagonistic parent-offspring co-adaptation does not require solicitation to be costly, allows for rapid divergence and evolutionary novelty and potentially explains the origin and diversification of the observed provisioning forms in family life.

## Introduction

Offspring influences on parental investment (PI) are ubiquitous [1]–[4]. For instance, mammal and plant embryos release hormones to sequester resources from maternal tissues [5], [6], insect juveniles emit chemical cues to which caregivers respond [7]–[9], and bird chicks display their characteristic begging displays (calling, wing-flapping) and morphologies (gapes, ornamental plumage [10], [11]). The evolution of offspring characters that influence PI is traditionally understood as the phenotypic, and usually costly, manifestation of resolved conflict between parents and their offspring over PI [1]–[4], [12], driven either by sibling rivalry [13] or parental preference [14]. The major predictions of conflict resolution theory have received experimental support overall [e.g.], [12,15], but it is also becoming increasingly clear that it does not capture a number of critical aspect in the co-evolution of offspring and parental behaviors [15]. A repeatedly mentioned shortcoming of this theory [15]–[21] is its static approach of modeling how “offspring individuals” and “parental individuals” should interact given conflict [but see; 22], [23].

Quantitative genetic models of maternal effects and parent-offspring interactions represent an alternative approach focusing on the maintenance of heritable (co-)variation by modeling how offspring and parent behaviors interact to determine offspring (or parental) fitness [19], [24]–[26]. This approach makes predictions for patterns of co-adaptation in terms of genetic correlations between offspring and parental traits based on selection acting on offspring or on parents, but it has not yet been incorporated into an explicit functional context for PI [27].

Here, we develop a dynamic evolutionary model based on co-evolving behavioral reaction norms [27], [28] and the premise of antagonistic selection on the amount of PI individuals *obtain/take* as offspring and the PI they provide as parents. This model is evaluated both deterministically and stochastically. Antagonistic selection occurs because, under a trade-off between the number and quality of offspring [29], [30], obtained PI enhances survival of individuals when they are offspring, but provided PI per offspring reduces fecundity of individuals when they are parents. We show that this antagonistic selection across life-stages generates a neutral space of equivalent co-adapted strategy sets individuals may express as offspring and as parents. This outcome allows for evolutionary innovation in family interactions by maintaining cryptic heritable variation and ongoing evolutionary dynamics in the behaviors mediating PI.

### The Model

We developed a co-adaptation model using both a deterministic analysis and stochastic simulations of antagonistic selection across life-stages. See Table 1 for a full list of definitions of model parameters and variables. The model assumes a trade-off between quality (survival) and number (fecundity) of offspring [29], [30], which necessarily implies that the amount of PI individuals obtain as an offspring tightly co-evolves with the amount provided as a parent (at equilibrium they have to be identical). In our model, selection on the offspring stage of generation *t* does not affect parental traits (generation *t*−1) because these individuals belong to different generations. Selection does however favor increased solicitation *x* in the offspring stage, which will increase the amount of obtained PI (*f_o_*) if, and only if, parents are sensitive. Conversely, selection on the parental stage of generation *t* can favor a reduction of provided PI (*f_p_*) in two ways: 1) by reducing the sensitivity *a* of a parent to offspring solicitation, or 2) by reducing a baseline amount *b* of PI. Differential selection on these two components of provided PI depends on the level of solicitation expressed in the offspring stage of generation *t*+1.

**Table 1:** Definitions of functions, model parameters and variables.

Variable/Parameter	Definition
General	
*f_o_*	Amount of PI obtained as offspring
*f_p_*	Amount of PI provided as parent to each offspring
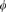	Population mean amount of PI
*t*	Time index, in generations (*t*→focal generation)
*S*()	Offspring survival function
*F*()	Parental fecundity function
*W*()	Lifetime individual fitness function
*H*()	Heaviside function (step-function to set survival to 0 if *f_o_*<*p*_min_)
*p*_min_	Minimum amount of PI required for offspring survival (fitness parameter)
*k*	Shape parameter for offspring survival function (fitness parameter)
*m*	Total PI available for reproduction (fitness parameter)
*a*	Parental sensitivity to offspring solicitation (evolving trait)
*x*	Offspring solicitation level (evolving trait)
*b*	Baseline level of PI (evolving trait)
Deterministic model	
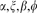	Population means (non-equilibrium) for parental sensitivity, offspring solicitation, baseline PI and total PI, respectively
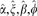	Equilibrium population means for sensitivity, solicitation, baseline PI and total PI, respectively
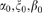	Initial values (generation 0) for population mean sensitivity, solicitation and baseline PI, respectively, in numerical analyses
Stochastic simulation	
*a_j_*	Sensitivity alleles 1–5 (stochastic simulations); *a*_1_→insensitive
*x_i_*	Solicitation alleles 1–5 (stochastic simulations); *x*_1_→no solicitation
*b_const_*	Baseline provided PI; fixed parameter

**Figure 1:**
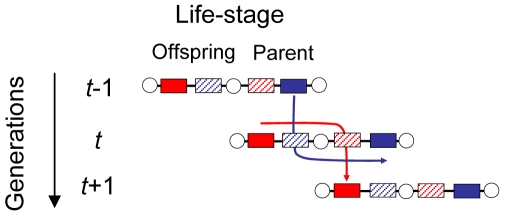
Transmission dynamics of provisioning genes (blue boxes) and solicitation genes (red boxes). Filled boxes indicate the gene is expressed in that life-stage, hatched boxes that the gene in not expressed. Arrows depict the path through which genes get passed on to the next generation.

Critically, a successful offspring solicitation strategy increases its representation in the parental stage of the current generation, but has yet to spread to the next generation. Thus, the spread of solicitation strategy *x* also depends on how the individual performs as a parent in terms of its fecundity and, hence, its sensitivity *a* and baseline PI *b* (Figure 1). Obviously, solicitation is not directly expressed during the parental life-stage, but, if the individual becomes a parent who is sensitive to offspring solicitation, there is an indirect genetic effect [31] of solicitation on *f_p_* [32] generating intergenomic (“social”) epistasis for fitness between offspring and parents [25], [33], [34]. Analogously, parental provisioning genes, despite only being expressed during the parental stage, must survive the offspring stage before they generate a fecundity pay-off during the stage at which they are expressed (Figure 1). Their evolutionary success depends on an interaction with the solicitation strategy *x* inherited to the offspring, to which they are also predictably associated within an individual (or genome). In summary, solicitation and provisioning co-evolve as interacting phenotypes [31], generate intergenomic (“social”) epistasis for fitness [25], [33], [34] and are under antagonistic selection across life-stages.

We assume throughout that a single parent interacts with a single offspring or, equivalently, with an arbitrary number of offspring that do not interact or compete. At evolutionary equilibrium, population means for *f_o_* and *f_p_* (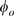 and 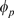 respectively), as mediated by the interaction of mean levels for solicitation 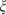, sensitivity 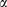 and baseline provisioning 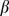, must be identical (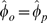). This equilibrium level of obtained/provided PI is denoted by 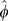 (Table 1).

### Deterministic Model

The survival during the offspring stage in relation to *f_o_* of individuals from target generation *t* is determined by a diminishing returns function like in traditional life-history and conflict resolution models [3], [4], [12], [29]. Specifically,
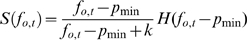
(1)where *p*_min_ is the minimal PI necessary for offspring survival and *k* is a ‘shape parameter’ determining the marginal returns on PI (Table 1). The Heaviside function (*H*) is a standard step-function used to set survival to zero if 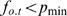 (i.e., to prevent negative fitness values). An important aspect of our model is that solicitation has no direct fitness cost during the offspring stage.

**Figure 2:**
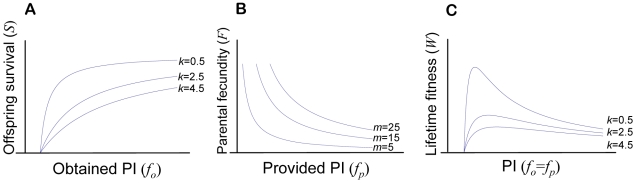
Fitness model used throughout. Panel A depicts how survival *S* is determined by the amount of obtained parental investment (*f_o,t_*) (for different shape parameters *k*), B how parental fecundity *F* changes with the amount of provided PI (*f_p,t_*) (for different total resources available for reproduction; parameter *m*), and C how individual lifetime fitness *W* changes in relation to PI when *f_o_* = *f_p_* and for different values of *k*.

If an individual of target generation *t* survived the offspring stage, fecundity during the parental stage was defined as a decay function depicting a fecundity cost of increased provided PI
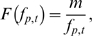
(2)where *m* is a fixed amount of resources available for lifetime reproduction [3], [4]. The assumption of a fixed *m* implies that obtained PI affects only offspring survival, not the resources available for reproduction when the individual becomes a parent. See Figure 2 for a graphical illustration of the fitness model and its components.

We used a quantitative genetic model of trait expression, incorporating the cross-generational interaction between parents and their offspring as an indirect genetic effect [31], [35] mediated by an evolving linear behavioral reaction norm [27]. The standard assumptions of quantitative genetic models apply (normally distributed trait values, random mating, constant additive genetic (co-)variances, and weak selection). We focused on the directional selection driving antagonistic co-adaptation in terms of evolving population mean levels of offspring solicitation and parental provisioning. Non-linear selection affects the genetic variances and co-variances, an aspect studied in previous co-adaptation models [24], [25], but it has no direct effect on evolving mean trait values [36] and thus was not included in the present analyses.

For simplicity, we assume PI responds linearly to solicitation according to linear behavioral reaction norms [27], [28]: *f_o,t_* = *a_t_*_−1_ *x_t_*+*b_t_*_−1_ and *f_p,t_* = *a_t_ x_t+_*_1_+*b_t_* (subscripts *t*−1 and *t*+1 refer to the previous and subsequent generation, respectively). To ensure behavioral stability and convergence of interacting offspring and parental behavioral reaction norms [37], and for tractability, we assume that a strategic change in provisioning has fitness consequences for, but no behavioral effect on, solicitation.

Fitness was calculated over both life-stages as the product of survival at the offspring stage and fecundity at the parental stage (i.e., *W* = *S*×*F*).
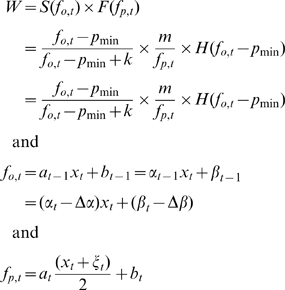
(3)where Greek versions of letters reflect the population mean for a trait, and the 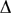 notation indicates the evolutionary change between generations *t*−1 and *t*. In order to get equation 3 we assume random mating, traits are additively inherited, that provisioning is performed uniparentally and the offspring is the caring parent's progeny. To calculate the average fitness function 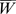, we assumed that the per generation changes in baseline provisioning and parental sensitivity are small relative to the mean values of these traits (i.e., 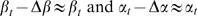).

Using the fitness function (*W*) from equation 3, we calculated the evolutionary dynamics of the system using the standard machinery of quantitative genetics [36]. In particular, we analyzed the dynamic system 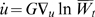 – where 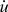 is the time derivative of *u* which is the trait vector {*α, ξ, β*} and 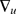 is the gradient operator – for equilibria and their stability. A first-order Taylor series approximation of *W* about the point 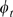, the mean of *f_o,t_* (equation 1), was used to compute the average fitness function
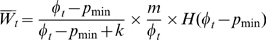
(4)


Because the evolutionary system is a gradient system, the single fitness maximum at 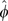 (Figure 2C) is an evolutionarily stable equilibrium of the system [36].

The time-dynamics of the system were numerically explored. Initial values for population mean parental sensitivity *α*_0,_ offspring begging *ξ*_0_, and baseline provisioning *β*_0_ for a starting population of interacting offspring and their parents were chosen such that the resulting level of PI was not too far from the equilibrium (±2 units of PI). This choice was made due to convergence problems when the dynamic system was started with more extreme values, and our analyses is therefore not for global stability (rather for an ‘attraction basin’); in practice, the only circumstance under which the system did not converge was when one of the three provisioning traits evolved to zero prior to achieving equilibrium, which was more likely to happen for extreme starting values. Numerical analyses were carried out using the software Mathematica (v. 6.0) [38].

### Stochastic Simulations

To address the role of stochastic effects in the antagonistic co-adaptation process, we developed a stochastic, numerical analogue of our deterministic model that explicitly incorporated mutation, migration and environmental stochasticity. The simulations were set up as a discrete-time Monte Carlo simulation model in which each event (survival, reproduction and death) occurred probabilistically. Offspring solicitation and parental sensitivity were represented by two distinct loci with alleles determining the corresponding levels of behavior. We assumed solicitation (*x*) was governed by one locus with *i* alleles and parental sensitivity (*a*) by one locus with *j* alleles. We assumed equal allele numbers for the two loci, and present the results for *i* = *j* = 5 (qualitatively similar results were obtained with different allele numbers; SG and MK unpublished results). The baseline provisioning *b_const_* was incorporated as a fixed parameter in the stochastic simulations. To parallel the analytical version of our model, we assumed PI responds linearly to solicitation. Each offspring genotype *x_i_* expressed a different level of solicitation, the lowest (*x*_1_) always being “no solicitation”; similarly, each parental genotype *a_j_* expressed a different level of sensitivity to solicitation, the lowest (*a*_1_) always being “no sensitivity”. The highest level (*x*_5_) for an allele was calculated as the one that, combined with the highest parental sensitivity (*a*_5_), would result in the upper limit for PI available to parents (parameter *m*). Alleles for intermediate solicitation levels (*x*_2_–*x*_4_) and intermediate sensitivity levels (*a*_2_−*a*_4_) were chosen to be evenly distributed between the minimum and maximum values.

We assumed a population composed of *n*/2 families (i.e., parent-offspring interactions) where *n* is the population size in terms of individuals present at any given moment in time; each family was composed of one parent and one offspring (i.e., individuals from successive generations). Individuals in a given life-stage have discrete and non-overlapping generations, but interact across life-stages and generations to determine PI. We further assumed the individuals are haploid and reproduce asexually. The haploid mode of inheritance was chosen for its simplicity. Nevertheless, it directly relates to the assumed additive mode of inheritance in the analytical version of the model because the expressed genetic variance under haploid inheritance is caused by additive components only [39, p. 92] (except for within-genomic epistatic interactions). To avoid some of the pitfalls of asexual reproduction in such simulations (e.g., lock-in of sub-optimal genotypes due to lack of genetic variability and sexual recombination), a relatively high mutation rate was chosen. Mutations caused a change of allelic state to another allele within the set of possible alleles. The mean±SE per-generation mutation rates over 1000 time-steps were 0.1004±0.0002 and 0.09873±0.0002 for the solicitation and sensitivity locus, respectively. The mutation rate was not supposed to reflect a biologically realistic rate, but rather to continuously regenerate heritable variation for parental sensitivity and offspring solicitation on which antagonistic selection could act in the absence of sexual recombination.

Similar to the analytical model, the success of an individual in generation *t* was the product of its survival *S* at the offspring stage and its fecundity *F* at the parent stage (see Table 1). Generation *t* was composed of two time-steps T in the simulation model (*t* = 2T), reflecting an offspring and parental life stage, respectively. The frequency of the solicitation genotype *x_i_* in the offspring subpopulation was computed at each time step T. Offspring survival/death happened with a likelihood proportional to *S*(*f_o,t_*). Specifically, the survival probability of an offspring carrying solicitation allele *x_i_* obtaining PI from its parent with sensitivity allele *a_j_*, was given by
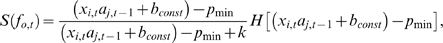
where 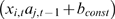 corresponds to the amount of obtained PI (*f_o,t_*). See table 1 for variable and parameter definitions. An offspring survived in the simulations if the value for *S*(*f_o,t_*) was larger than a random number drawn from a uniform distribution.

Equivalently, reproduction happened with a likelihood proportional to *F*(*f_p,t_*) the fecundity of a parent carrying sensitivity allele *a_j_* that provides PI to its offspring with solicitation allele *x_i_*. The frequency of the parental sensitivity genotype *a_j_* in the offspring sub-population of individuals from the next generation (*t*+1) was assumed to evolve according to
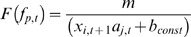
where 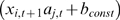 corresponds to provided PI (*f_p,t_*) as determined by the interaction of the sensitivity allele *a_j,t_* of the focal generation individuals in their parental stage, and the solicitation genotype of their offspring (*x_i,t_*_+1,_). As before for survival, a parent reproduced if the value for *F*(*f_p,t_*) was larger than a random number drawn from a uniform distribution.

The population was assumed to be of constant size (in the examples shown, the population size *n* was equal 1000). To prevent population extinction we incorporated a stochastic immigration process that compensated for offspring mortality and reproductive failure. The genotypes of immigrant individuals were assumed to occur at a likelihood proportional to the genotype frequencies present in the resident population (i.e., we assumed that only genotypes similar to the ones established could successfully immigrate).

To test the prediction that the evolved offspring solicitation strategy should depend on the evolved parental sensitivity strategy (expressed during the adult stage), we ran simulations with fixed parental sensitivity strategies (*a*_1_−*a*_5_). To test stochastic influences for the evolution offspring solicitation and parental sensitivity, we ran simulations where offspring solicitation and parental sensitivity could both mutate and co-evolve. All simulations started with no genetic variability in the population; specifically all individuals carried the non-soliciting allele (*x*_1_) for the offspring strategy and the insensitive allele (*a*_1_) for the parental strategy. We used an experimental design with 100 replicate simulations per sensitivity level, and 1000 time steps (500 generations) per run.

### Model Results

#### Deterministic model

In this model, 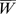
(equation 4) has one fitness-maximizing equilibrium with respect to PI at 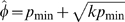, which was found by setting 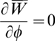 and solving for 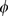. This equilibrium can be shown to always be at least neutrally stable with respect to population means for parental sensitivity *α*, offspring solicitation *ξ* and baseline provisioning *β*, such that
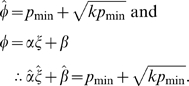
(5)


**Figure 3:**
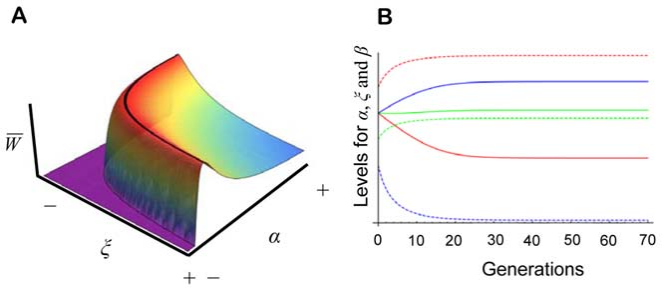
Co-adaptation equilibrium and neutral stability. In A the fitness surface displaying the neutral curve for co-adapted associations of offspring solicitation and parental sensitivity for a fixed value of *β* is shown. B illustrates a typical dynamics of the system from two different sets of initial conditions (solid versus dashed lines). Red lines are for parental sensitivity *α*, green lines for offspring solicitation *ξ* and blue lines for the baseline provisioning *β*; clearly, under different initial conditions the system reaches different stable equilibria that are part of the stable equilibrium ‘curve’ in A.

This equilibrium defines combinations of parental sensitivity, baseline provisioning and offspring solicitation within individuals (genomes) due to antagonistic selection across life-stages, and we refer to it as a co-adaptation equilibrium (Figure 3). It can be further shown by numerical analysis that the dynamic system reaches the co-adaptation PI equilibrium (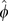) irrespective of the structure of the genetic covariance matrix, i.e., also in the presence of genetic correlations between *a*, *x* and *b* (an analytical proof is available upon request as well).

The numerical analysis of the deterministic system was carried out systematically with parameter values *p*_min_ = 2, *k* = 2.5, *m* = 10, yielding an optimum level for 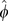 of approximately 4.2361. These are the same parameter values as those used in our stochastic simulations (see below). Different sets of genetic variances and genetic correlations among the traits were explored, with initial values (*t* = 0) for each trait (*α*_0_, *ξ*_0_, *β*_0_) , genetic variances for, and correlations among traits chosen at random to numerically explore the stability of the co-adaptation equilibrium for different genetic covariance matrices. In all cases, the system evolved to the expected value for 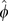 of 4.2361, irrespective of the genetic variances and correlations among the traits. This result holds for many more conditions of covariance matrices and initial trait values (BJR and MK unpublished results) as long as none of the three trait values evolved to zero prior to achieving equilibrium, a condition causing convergence problems.

### Stochastic Simulations

Based on the results of the analytical model (Figure 3), we predicted that 1) successful invasion and maintenance of offspring solicitation strategies should be critically determined by the provisioning strategy (sensitivity) expressed at the parental stage of the individuals in a population (co-adaptation), and 2) stochastic effects may lead to transitions in offspring solicitation and parental provisioning strategies within the limits set by the co-adaptation equilibrium without altering PI (neutral curve). Thus, a stable level of PI should be maintained despite ongoing changes at the genetic level for solicitation and sensitivity.

**Figure 4:**
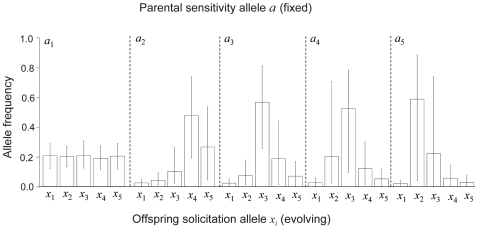
Simulation outcomes with parental sensitivity fixed. *a*_1_ represents an insensitive parental strategy, and *a*_5_ the most sensitive parental strategy. The solicitation locus is allowed to mutate among the five alleles and evolve. *x*_1_ is a non-soliciting strategy, *x*_5_ the highest solicitation strategy. and adapt to the parental sensitivity present in the parental sub-population. The mean and 95% percentiles of the distribution of evolutionary outcomes are shown for the frequencies of each solicitation allele after 500 generations.

The first prediction was tested in simulations where the parental allele was fixed at different levels of sensitivity (*a*_1_−*a*_5_), and the solicitation locus was allowed to evolve and adapt to the fixed parental sensitivity. As predicted, the successful spread of different offspring solicitation strategies depended on the particular fixed sensitivity allele present in the parental stage of individuals of the population (ANOVA: interaction sensitivity allele x solicitation allele, *F*_16,2475_ = 224.43, *p*<0.001; Figure 4). With insensitive parents, offspring solicitation is selectively neutral in our model and, correspondingly, solicitation strategies from low to high emerged and were maintained at low and similar frequencies. When a sensitive parental provisioning allele was fixed, a particular solicitation strategy quickly invaded and evolved to high frequency, but which solicitation allele took over depended on the parental sensitivity genotype. With high parental sensitivity, the low-solicitation alleles occurred at higher frequencies; similarly if parents were less sensitive, then high-solicitation alleles occurred at greater frequency (Figure 4).

**Figure 5:**
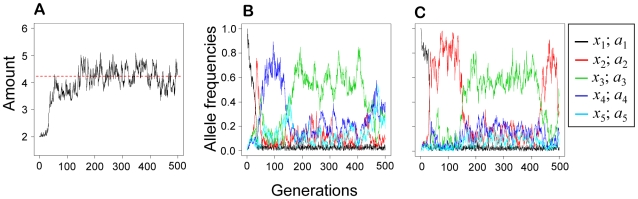
Simulation outcome with offspring solicitation and parental sensitivity co-evolving. Example of a single simulation run. Panel A) shows the evolution of the population mean level of PI (the dashed red line reflects the theoretical optimum level of PI for the chosen parameter values). B) the evolving frequencies of the five solicitation alleles, and C) the evolving frequencies of the five sensitivity alleles.

**Figure 6:**
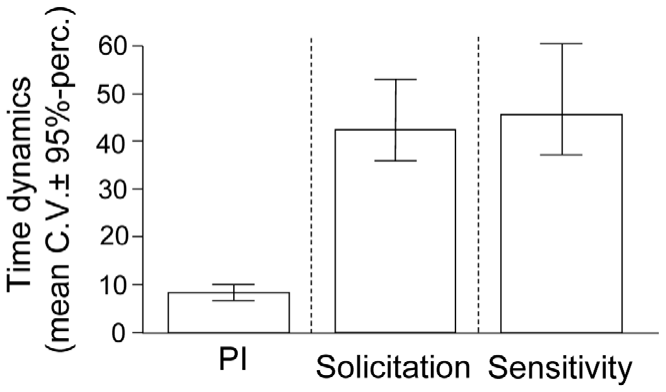
Time dynamics in the stochastic simulations. The co-evolutionary time-dynamics for the level of PI, solicitation allele frequencies and sensitivity allele frequencies. Shown are the mean and 95% percentiles for the coefficients of variation (C.V.) calculated over the final 250 generations and across 100 simulation runs.

To test the second prediction, offspring and parental strategies were both allowed to evolve and co-adapt. As expected, co-evolutionary transitions sometimes occurred after PI reached the expected optimum (Figure 5B,C), and these transitions were barely detectable in terms of PI (Figure 5A). To quantitatively show evolutionary stasis in PI despite ongoing dynamics in offspring solicitation and parental sensitivity, we compared the coefficients of variation (C.V.) over time (second half of the simulations = 250 generations) between the evolving PI-level and the evolving solicitation and sensitivity allele-frequencies. The average CV and 95%-percentiles were computed across the 100 replicate runs. As expected, there was little variation in PI (less than 10%), but around 40% variation in solicitation and sensitivity allele-frequencies (Figure 6). This result confirms that a relatively stable optimal level of PI can be maintained by a range of co-adapted offspring and parental strategy sets that are equivalent in terms of lifetime fitness, just as our analytical result of neutral stability predicts.

## Discussion

Our model demonstrates that antagonistic selection across the offspring and parental life-stage of individuals favors the expression of co-adapted strategies that match to maximize an individual's lifetime fitness. In our model, offspring solicitation has no direct cost during the offspring stage and emerges from arbitrary cues as an adaptation to emerging parental sensitivities rather than as a resolution of parent-offspring conflict. We propose that under a trade-off between the number and quality of offspring (which is well supported experimentally; e.g. [40]) antagonistic co-adaptation is a critical component shaping offspring and parental strategies in the evolution of family interactions. It is an alternative, albeit not mutually exclusive, hypothesis for the evolution of offspring solicitation focused on co-evolving and interacting behavioral reaction norms [27], the resulting intergenomic epistasis for fitness and the co-adaptation of offspring and parental strategies within genomes.

The “possibility of coadaptive evolution”[16] in parent-offspring interactions was pointed out for the first time by Feldmann and Eshel [16], but explicit co-adaptation models were developed much later [24]–[26]. These previous co-adaptation models predict adaptive genetic correlation between offspring and parental traits due to maternal effects [24] or parent-offspring interactions [25] leading to the co-inheritance of offspring and parental behaviors. There is increasing experimental evidence supporting the prediction for co-inherited offspring and parental behaviors (see [25], [27], but see [41]), which implies that genes underlying offspring and parental behaviors may often segregate non-randomly and tend to be co-inherited. These models focused on the evolution of genetic correlations through linkage disequilibrium given offspring solicit and parents respond. They assumed selection to act either on offspring [24]–[26] or on parents [25], and that the interaction coefficients were evolutionarily fixed (i.e., constant strengths of indirect genetic effects). The present model differs from these previous co-adaptation models in that it derives a co-adaptation equilibrium in terms of population mean offspring and parental behaviors in an explicit functional context of PI (i.e., antagonistic selection across life-stages), tests the potential consequences of genetic correlation on the stability of this equilibrium and allows the parental behavioral reaction norm to evolve fully in terms of both sensitivity to solicitation and baseline provisioning.

The co-adaptation equilibrium 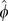 (equation 5) has several interesting properties. First, and similar to conflict resolution models, offspring solicitation, parental sensitivity, and baseline provisioning depend upon the marginal return on PI in terms of offspring survival (as determined by *k*) [4], [14]. Second, the genetic covariance between solicitation and sensitivity do not influence the equilibrium values for the strategies, and, contrary to expectations from previous suggestions [33], [42], [43], do not destabilize the equilibrium level of PI by, for example, triggering a runaway process. Third, the co-adaptation equilibrium is actually a ‘curve’ in space (Figure 3A) with many equilibria, including non-soliciting offspring and insensitive parents. Thus, the actual equilibrium trait values depend upon initial conditions of the system (Figure 3B) and, because the equilibrium is neutrally stable, the system can ‘drift’ to different points along the equilibrium curve. If the system is perturbed after reaching equilibrium it may not return to the identical equilibrium values depending on the extent of this perturbation (Figure 5). This last result suggests evolutionary robustness [44] in PI and an important role for stochastic evolutionary processes, such as mutation or migration, and environmental fluctuations facilitating the original evolution of offspring solicitation and parental sensitivity [15], [20]. Such events may represent instances of evolutionary innovation such as, for example, the origin of offspring solicitation. Thus, while PI itself is expected to be robust evolutionarily, the underlying traits used to mediate its provisioning should be highly labile and maintain substantial cryptic heritable variation and evolvability ([44], [45]; see also [24], [25]). As a result, populations and/or species may diverge and diversify quickly in how individuals solicit as offspring and respond as parents [46], despite similar levels of PI.

This co-adapted space arises because it constitutes “a collection of equivalent solutions to the same biological problem” [44; p. 195], the problem being in this case obtaining (as an offspring) and providing (as a parent) an amount of parental investment which maximizes lifetime fitness. All else being equal, lifetime fitness can be the same in rigid investment systems where parents simply provide an optimal baseline investment (i.e. 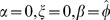) or communicative systems (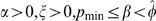). Only once parents evolve sensitivity to an otherwise selectively neutral offspring cue, can the evolution of an offspring signal originate [47]. Parental sensitivity may emerge purely stochastically as a sensory bias [48] (like in our model), or as an adaptation if sensitive parents provide resources more effectively [49], [50]. It will be interesting to extend our model to incorporate a direct benefit to sensitive parental strategies and a direct cost to offspring solicitation.

We assumed throughout that soliciting is not costly to offspring. If antagonistic co-adaptation drives the evolution of offspring solicitation, begging costs during the offspring stage are not necessary for evolutionary stability, and the evolved (neutrally stable) solicitation level is determined by the sensitivity expressed in the parental stage. This stabilizing effect is due to the indirect genetic effect of solicitation on PI which leads to fecundity losses in individuals who are sensitive parents [32]. Thus, under antagonistic co-adaptation, intense solicitation does not automatically imply costliness or selfishness, which may help explain why many offspring cues or signals to which parents respond are subtle in nature [51], and begging costs to offspring – even for the vigorous bird begging displays – are often surprisingly low (see [11] and chapters therein).

The major conclusions of our model are that antagonistic co-adaptation is the direct consequence of an offspring quality-quantity trade-off generating selection on both solicitation and provisioning genes that temporarily share a genome when expressed in different life-stages of individuals, and interact across generations in a predictable manner (i.e., as determined by the degree of genetic relatedness). While our model is on the evolution of parent-offspring interactions and offspring solicitation, antagonistic selection across the offspring and parental life-stage can play an important role in life-history evolution in general, whenever trade-offs differentially affect fitness components to the offspring versus the parental life-stage. For instance, data from a long-term field study in soay sheep (*Ovis aries*) showed that selection favored singleton births at the offspring stage, but twin births at the parental stage [52]. Considering the consequences of antagonistic co-adaptation in the evolution of parent-offspring interactions might change predictions of conflict resolution theory, which assumed independent segregation of offspring and parental strategies [3], by, for example, constraining the evolutionary success of selfish and costly offspring strategies [16], [19], [32]. One recently suggested alternative possibility [26] is that different sets of genes may be shaped by co-adaptation and conflict. Genes under selection from co-adaptation would ensure the functional integrity of the parent-offspring interaction over the lifetime of individuals, while other loci under selection from conflict may lead to the evolutionary exaggeration of the signals due to a competitive advantage during the offspring life-stage. In future efforts to unify the evolutionary theory underlying family interactions, researchers need to clarify exactly how conflict resolution and co-adaptation relate to each other [27].
